# Metolazone Add-On Therapy in Heart Failure: A Cohort Study from Persian Registry of Cardiovascular Disease/Heart Failure (PROVE/HF)

**DOI:** 10.1155/2021/3820292

**Published:** 2021-10-22

**Authors:** Farzad Rahimi, Mehrbod Vakhshoori, Maryam Heidarpour, Fatemeh Nouri, Kiyan Heshmat-Ghahdarijani, Mohammad Fakhrolmobasheri, Davood Shafie

**Affiliations:** ^1^Heart Failure Research Center, Cardiovascular Research Institute, Isfahan University of Medical Sciences, Isfahan, Iran; ^2^Isfahan Endocrine and Metabolism Research Center, Isfahan University of Medical Sciences, Isfahan, Iran; ^3^Isfahan Cardiovascular Research Center, Cardiovascular Research Institute, Isfahan University of Medical Sciences, Isfahan, Iran

## Abstract

**Background:**

One of the strategies for overcoming diuretic resistance among heart failure (HF) patients is adding thiazide-type diuretics. The main aim of this article is to compare the adverse clinical outcomes, including death and re-hospitalization, among individuals suffering from severe acute decompensated HF (ADHF) that consumed furosemide or furosemide plus metolazone.

**Methods:**

This retrospective cohort study was done in the context of the Persian registry of cardiovascular disease (PROVE) from September 2017 to September 2018. One thousand and four hundred thirty-eight individuals (furosemide: 972 and furosemide plus metolazone: 466) with the final diagnosis of severe ADHF (left ventricular ejection fraction < 30%) were selected and followed for 10.3 ± 7.8 months. The association between two groups, as mentioned above, with the incidence of death and re-admission, was evaluated with different models.

**Results:**

The mean age of the study population was 68.19 ± 12.98 years. There was no significant relation in terms of death or re-hospitalization between patients with different diuretic regimens. After adjustment of potential confounders, we found that adding metolazone as an adjuvant HF therapy was not independently associated with death or re-hospitalization (hazard ratio (HR): 0.78,95% confidence interval (CI) = 0.59–1.03, *P* = 0.085, and odds ratio (OR): 0.80, 95% CI: 0.60–1.07, *P* = 0.135, respectively).

**Conclusion:**

Our findings revealed that adding metolazone in patients with furosemide resistance is not associated with higher morbidity and mortality. Therefore, usage of these two therapeutic agents could be a helpful strategy for severe HF patients.

## 1. Introduction

Increasing the prevalence of previously proved cardiovascular risk factors among developed and developing nations leads to categorizing cardiovascular diseases (CVDs) as the leading cause of mortality [1–6]. One of the most debilitating diseases is heart failure (HF), defined as a declining cardiac pump function to meet tissue metabolic demands. Although several prognostic factors, as well as therapeutic methods, have been suggested till now, this chronic disorder has been associated with 31.7% mortality. It causes $ 108 billion dollars for its annual management cost [7–10]. For each patient, this economic burden ranges from $ 908 to $ 40,971, and it has been reported that more than $ 50 billion would be expended for acute decompensated HF (ADHF) by the year 2030 [2, 11]. Fluid overload remains one of the main obstacles among HF patients; therefore, providing a euvolemic state is mandatory, which could be done with several strategies. Despite no approved superiority in terms of survival rate with loop diuretics, these agents remain the cornerstone part of HF treatment in a way that this drug had been prescribed for 86–97% of individuals hospitalized for ADHF [11, 12]. By the way, the efficacy of the agents mentioned above is not optimal among some patients leading to the introduction of a term named “diuretic resistance (DR).” Several definitions have been described, and some of them include persistent overload symptoms despite > 80 mg of daily furosemide, excretion of less than 90 mmol of sodium in the presence of 160 mg furosemide during three days, or less than 0.2% sodium excreted as filtered load [13, 14]. Although the exact pathophysiological mechanism for this phenomenon has yet to be defined, drug interaction, increased neurohormonal activity plus reduced drug delivery to its proper site of action, and compensatory adaptation in tubular cells are proposed as probable explanations [15]. On the other hand, several interventions have been suggested to overcome this resistance; some include salt restriction, heightened loop diuretic dosage, or adding a different class of diuretic agents [15, 16]. Metolazone (7-chloro-2-methyl-3-(2-methyl phenyl)-4-oxo-2,3-dihydro-1H-quinazoline-6-sulfonamide) was first introduced in the 1970s, and it has been categorized as a potent agent in declining diuretic drug resistance due to desirable properties like lower cost and higher bioavailability [16, 17]. Although diuretic resistance has been proved to be associated with higher mortality, long-term complication incidence in the presence of metolazone as adjuvant therapy is less frequently investigated [18].

This article sought to assess the mortality and re-hospitalization rates among patients suffering from ADHF with/without metolazone add-on therapy.

## 2. Materials and Methods

### 2.1. Study Population

This retrospective cohort study was conducted in the context of the Persian registry of cardiovascular disease/HF (PROVE/HF) project. In brief, the main aim of the latter ongoing study was developing a database to implement as a national guideline for the assessment of information about prevention, diagnosis, and treatment of CVDs [19, 20]. From September 2017 to September 2018, any patients admitted with the diagnosis of severe ADHF (left ventricular ejection fraction (LVEF) < 30%) were eligible for recruitment in this study. Being under 18 years or unwilling to participate in the study as well as incompleteness of data profile were defined as exclusion criteria. The participants were divided into two groups according to the diuretic agent prescription categories (furosemide and furosemide plus metolazone group) prescribed at the discharge date, and the occurrence of death and re-hospitalization was assessed during the follow-up duration. The purpose of the study was explained to each individual by the principal investigator, and all participants had sufficient time to ask any probable questions. They were also allowed freely to leave the study at any time without any further consequences. After implementing all inclusion and exclusion criteria, 1438 individuals (furosemide group: 972 and furosemide plus metolazone group: 466) were eligible for recruitment, and their whole data were analyzed. This study was approved by the ethics committee of the Isfahan University of Medical Sciences (IR.MUI.REC.1396.3.105).

### 2.2. Assessment of Variables

Each subject's medical form was gathered for age, sex (male/female), body mass index (BMI), and smoking status. Moreover, data on the previous history of hypertension, diabetes mellitus, chronic obstructive pulmonary disease, ischemic heart disease, stroke, and renal problems were collected by a two-item scale (yes/no). Blood pressure indices, including systolic blood pressure (SBP) and diastolic blood pressure (DBP), as well as heart rates, were assessed during admission. Laboratory parameters, including hemoglobin, blood urea nitrogen (BUN), and creatinine (Cr), were assessed from patients' medical profiles. Data on medication usage, including angiotensin-converting enzyme inhibitors (ACEIs), angiotensin receptor blockers (ARBs), beta-blockers, mineralocorticoid receptor antagonists, nitrates, digoxin, and oral anticoagulants, were also assessed both in pre-admission and discharge dates. After discharge, each individual was followed by a telephone survey, and the probable occurrence of death and re-hospitalization was obtained. Participants were also followed for the consumption status of diuretics. In the case of the occurrence of our pre-defined outcomes, the patient or his/her relatives were invited to display the relevant documentation.

### 2.3. Statistical Analysis

Categorical and continuous variables were reported as frequency (percentage) and mean ± standard deviation (SD). Student's *t*-test and chi-square statistical examinations were utilized to assess the relation of numerical and nominal variables, respectively. Cox regression hazard ratio (HR) and odds ratio (OR) models were used to evaluate the relation of death and re-hospitalization based on the categories of diuretic agent usage, respectively, with univariate and multivariate models adjusted for age, sex, BMI, ischemic heart disease, diabetes mellitus, hypertension, stroke, kidney diseases, chronic obstructive pulmonary disease, smoking, SBP, DBP, heart rates, hemoglobin, sodium, potassium, BUN, Cr, and discharged drug consumption (beta-blockers, ACEIs, ARBs, mineralocorticoid receptor antagonists, digoxin, and nitrates). The multivariate model was used to assess the sole effect of diuretic agent groups on death and re-hospitalization. For evaluation of group differences among participants in groups of furosemide or furosemide plus metolazone based on death status, Kaplan–Meier curves with log-rank tests were used. Statistical Package for the Social Sciences (SPSS, version 22.0) was used to implement all analyses, and *P* values less than 0.05 were defined as statistically significant.

## 3. Results

**Figure 1:**
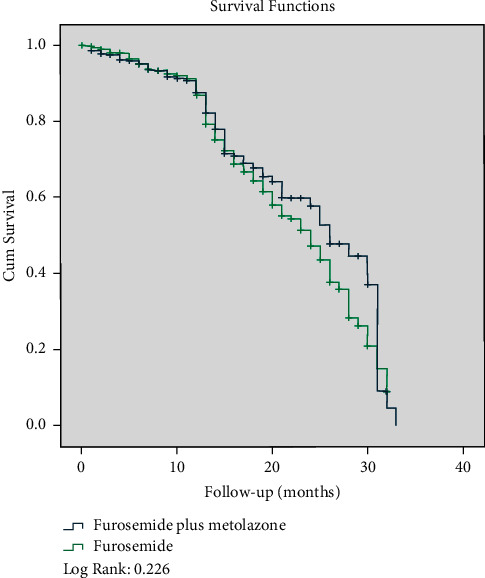
Survival curves for death according to different categories of diuretic agents.

The mean age of the study population at baseline was 68.19 ± 12.98 years. More than half of the study sample contained male participants. The total daily mean dosage of furosemide in patients consuming this diuretic was 122.4 ± 62.1 mg. In the second group, the total daily mean of furosemide and metolazone was 160.5 ± 38.8 mg and 5.2 ± 2.8 mg, respectively. Patients were followed for a mean of 10.3 ± 7.8 months. General characteristics of individuals according to the categories of diuretic usages at the baseline are shown in Table 1. Individuals consuming furosemide plus metolazone were mostly females and had lower hemoglobin levels than those taking furosemide (36.9% vs. 27.5%, *P* < 0.001, and 13.09 ± 2.09 g/dl vs. 13.50 ± 2.04 g/dl, *P* < 0.001, respectively). Beta-blockers and ACEIs/ARBs were mostly used by patients within the furosemide plus metolazone group before admission, with a remained difference after discharge. No significant relations were found regarding the history of chronic diseases in the pre-admission state. During the entire follow-up duration, 320 (22.3%) deaths and 378 (26.3%) re-admissions occurred.  Table 2 provides information about death and re-hospitalization status according to different diuretic usage categories. Our findings showed that there was no significant difference between death and re-hospitalization according to diuretic regimens (death: furosemide plus metolazone: 20.8% vs. furosemide: 22.9%, *P* = 0.364; re-hospitalization: furosemide plus metolazone: 24.2% vs. furosemide: 27.3%, *P* = 0.224). Data on HR and OR of our pre-defined complications based on different categories of diuretic usages are shown in Table 3. We found no significant relation in terms of death neither in univariate nor in multivariate models (HR: 0.86, 95% confidence interval (CI): 0.68–1.10, *P* = 0.241, and HR: 0.78, 95% CI: 0.59–1.03, *P* = 0.085, respectively). Patients that consumed furosemide and metolazone also had insignificant lower odds of re-hospitalization than the reference group (OR: 0.80, 95% CI: 0.60–1.07, *P* = 0.135). As depicted in Figure 1, Kaplan–Meier curves for death revealed that participants who used two diuretic agents had no remarkable different survival rates rather than individuals who used furosemide (*P* = 0.226).

## 4. Discussion

Our principle aim of the current study was to evaluate the probable occurrence of death and re-hospitalization among Iranian ADHF patients who consumed either furosemide or furosemide plus metolazone. Our findings revealed that adding metolazone as an adjunctive HF therapy was not associated with higher mortality or re-admission rates during the follow-up period. Therefore, adding the thiazide diuretic might be a reasonable approach while patients were experiencing DR. Till now, there is no study evaluating the probable effect of these factors on mortality and re-admission rates among ADHF patients receiving either furosemide or furosemide plus metolazone, and further studies are required in this regard.

To the best of our knowledge, there are few studies done in the literature concerning the main aim of the current article. Brisco-Bacik et al. implemented a prospective cohort study to investigate the outcomes of two ADHF therapeutic strategies, including metolazone addition or escalation of diuretic dosages. Three independent hospitals were selected to obtain data of admissions from January 2013 to September 2015. Of all 13898 admissions, 7.5% (*n* = 1048) of them had documentation proving that metolazone was added as adjunctive HF therapy. They followed their subjects for a median duration of 423 days to evaluate all-cause mortality. Their crude model revealed that patients who took adjunctive metolazone had higher HR of deaths (HR: 1.61, 95%CI: 1.42–1.83, *P* < 0.001). Further multivariable adjustment of confounders remained statistically significant (HR: 1.20, 95% CI: 1.04–1.39, *P* = 0.01). Likewise, patients who received higher diuretic dosages had heightened all-cause mortality risk in the crude model (HR: 1.16, 95%CI: 1.08–1.23, *P* < 0.001). In contrast, multivariable-adjusted models failed to prove any significant association in this regard (HR: 0.97, 95% CI: 0.90–1.06, *P* = 0.52). They also further analyzed the data of patients who received high-dose diuretics. Similar to our findings, their findings revealed that the addition of metolazone in case of high-dose diuretic therapy was not associated with higher mortality chances neither in crude nor in adjusted models (crude model: HR: 1.10, 95% CI: 0.73–1.39, *P* = 0.95, and adjusted model: HR: 0.73, 95% CI: 0.50–1.08, *P* = 0.11) [21].

Our findings revealed re-hospitalization was not different between patients who consumed furosemide plus metolazone versus furosemide. Likewise, Cox et al. implemented a randomized, double-blinded clinical trial. They enrolled 60 acute HF patients who suffered from DR and distributed them randomly to three arms (oral metolazone, IV chlorothiazide, and tolvaptan). They followed them for 30 days after discharge and found that the re-admission rate was not different in the metolazone add-on group compared to the others [22].

Most other studies done in literature mainly focused on short-term diuretic therapy outcomes during hospitalization, including sodium, potassium, and urine output. For instance, data analysis at a discharge date of ADHF patients revealed that adding metolazone to furosemide was associated with a higher prevalence of hypokalemia and hyponatremia and worsening of renal functions in comparison to patients using just furosemide (*P* < 0.0001) [21].

On the other hand, several studies suggested the safety of the aforementioned diuretic agent for considering as an add-on strategy. Shulenberger et al. designed a study to assess the efficacy of adding metolazone to 89 ADHF patients suffering from DR compared to those receiving hydrochlorothiazide (*n* = 88) during hospitalizations. They realized that metolazone adjunctive therapy showed similar outcomes in terms of renal function and electrolyte abnormalities with the latter agent [23]. Moreover, another cohort study done on 55 patients suffering from ADHF and concurrent renal dysfunction showed that there were no significant differences between 33 and 22 individuals being prescribed adjuvant metolazone or chlorothiazide therapy with loop diuretics in terms of sodium, potassium, hypotension, net urine output, and renal function worsening [24].

Due to a lack of sufficient studies in this regard, the exact explanation for these controversial results remains unknown. One possible theory could be that despite some adverse effects associated with metolazone usage, including hypokalemia and hyponatremia, this agent was not associated with higher mortality or re-admission rates. Therefore, this therapeutic strategy might be safely considered in patients with concurrent severe HF and DR.

The quite large sample size and a reasonable duration of follow-up were some strengths of this study. By the way, some limitations should be considered. We did not obtain information about the left ventricular end-diastolic volume of each participant, which might be effective on our outcomes. We did not assess any information about diuretic dosage alterations during the follow-up survey. We did not have information on ivabradine or angiotensin receptor neprilysin inhibitors for using in our data analysis, which might negatively affect our findings.

Furthermore, we performed this study in one city and with just one type of diuretic agent as add-on therapy, which necessitates further studies with other diuretic types. Therefore, the generalizability of the findings must be done with caution. Finally, we were unable to assess the post-discharge care status of the patients, probably accomplished by care managers or the patients themselves. The potential role of care managers in our final outcomes has been reported to be effective. Ciccone et al. implemented a team-based model named Project Leonardo to evaluate team collaboration's effectiveness in managing patients. They trained 30 care manager nurses to connect specialists, general practitioners, and patients suffering from HF, diabetes, and CVDs and performed this study for 18 months. They found that implementing the care manager model led to a positive impact on patients' health and self-management characteristics [25].

In conclusion, we found that metolazone add-on therapy during furosemide resistance would not be associated with higher mortality or re-hospitalization rates. This safe therapeutic intervention might be considered in clinical settings. Multiple comprehensive studies are required to prove these findings.

## Figures and Tables

**Table 1:** Characteristics of the study population across different categories of diuretic usages.

Variables		All (*n* = 1438)		Furosemide plus metolazone (*n* = 466)		Furosemide (*n* = 972)		*P*	
Age (years)		68.19 ± 12.98		67.79 ± 13.38		68.39 ± 12.79		0.411	
Male (%)		999 (69.5)		294 (63.1)		705 (72.5)		< 0.001	
BMI (kg/m2)		26.26 ± 4		26.46 ± 4.7		26.17 ± 3.6		0.193	
Hypertension (%)		911 (63.4)		303 (65)		608 (62.6)		0.363	
Diabetes mellitus (%)		675 (46.9)		218 (46.8)		457 (47)		0.933	
COPD (%)		175 (12.2)		46 (9.9)		129 (13.3)		0.065	
Ischemic heart disease (%)		1218 (84.7)		389 (83.5)		829 (85.3)		0.372	
Stroke (%)		74 (5.1)		22 (4.7)		52 (5.3)		0.613	
Renal diseases(%)		353 (24.5)		117 (25.1)		236 (24.3)		0.733	
Smoking (%)		265 (18.4)		81 (17.4)		184 (18.9)		0.479	
Systolic blood pressure (mmHg)		127.60 ± 26.3		127.56 ± 26.9		127.62 ± 25.9		0.965	
Diastolic blood pressure (mmHg)		80.67 ± 15.7		80.01 ± 15.6		80.98 ± 15.8		0.273	
Heart rates (beats/min)		89.22 ± 19.8		88.96 ± 19.6		89.35 ± 19.9		0.728	
Hb (g/dl)		13.37 ± 2.06		13.09 ± 2.09		13.50 ± 2.04		< 0.001	
BUN (mg/dl)		27.88 ± 14.40		28.62 ± 16.04		27.52 ± 13.5		0.175	
Cr (mg/dl)		1.51 ± 0.8		1.52 ± 0.7		1.50 ± 0.8		0.653	
Potassium (mEq/l)		4.49 ± 0.6		4.43 ± 0.6		4.52 ± 0.6		0.015	
Sodium (mEq/l)		138.66 ± 5		138.62 ± 5.1		138.68 ± 4.9		0.819	

Drug history									
Pre-admission	ACEIs/ARBs (%)		1021 (71)		418 (89.7)		603 (62)		< 0.001
	Beta-blockers (%)		1040 (72.3)		437 (93.8)		603 (62)		< 0.001
	Mineralocorticoid receptor antagonists (%)		468 (32.5)		159 (34.1)		309 (31.8)		0.377
	Nitrates (%)		665 (46.2)		206 (44.2)		459 (47.2)		0.283
	Digoxin (%)		465 (32.3)		156 (33.5)		309 (31.8)		0.522
	Oral anticoagulants (%)		287 (20)		102 (21.9)		185 (19)		0.205

Discharge	ACEIs/ARBs (%)		1211 (84.2)		457 (98.1)		754 (77.6)		< 0.001
	Beta-blockers (%)		1097 (76.3)		457 (98.1)		640 (65.8)		< 0.001
	Mineralocorticoid receptor antagonists (%)		770 (53.5)		262 (56.2)		508 (52.3)		0.159
	Nitrates (%)		820 (57)		265 (56.9)		555 (57.1)		0.934
	Digoxin (%)		735 (51.1)		237 (50.9)		498 (51.2)		0.894
	Oral anticoagulants (%)		345 (24)		126 (27)		219 (22.5)		0.061

BMI: body mass index, COPD: chronic obstructive pulmonary disease, Hb: hemoglobin, BUN: blood urea nitrogen, Cr: creatinine, ACEIs: angiotensin-converting enzyme inhibitors, and ARB: angiotensin receptor blockers.

**Table 2:** Distribution of death and re-hospitalization across different categories of diuretic usages.

Complications	All (*n* = 1438)	Furosemide plus metolazone (*n* = 466)	Furosemide (*n* = 972)	*P*
Death (%)	320 (22.3)	97 (20.8)	223 (22.9)	0.364
Re-hospitalization (%)	378 (26.3)	113 (24.2)	265 (27.3)	0.224

**Table 3:** Hazard ratio and odds ratio of death and re-hospitalization among study population according to different categories of diuretic usages.

Variables	Models	Diuretic agents		*P*
		Furosemide	Furosemide plus metolazone	
Death	Univariate	1.00	0.86 (0.68–1.10)	0.241
	Multivariate ^*∗*^	1.00	0.78 (0.59–1.03)	0.085

Re-hospitalization	Univariate	1.00	0.85 (0.66–1.10)	0.224
	Multivariate ^*∗*^	1.00	0.80 (0.60–1.07)	0.135

^*∗*^Adjusted for age, sex, body mass index, ischemic heart disease, diabetes mellitus, hypertension, stroke, kidney diseases, chronic obstructive pulmonary disease, smoking, systolic blood pressure, diastolic blood pressure, heart rate, hemoglobin, sodium, potassium, blood urea nitrogen, creatinine, and discharged drug consumption (beta-blockers, angiotensin-converting enzyme inhibitors, angiotensin receptor blockers, mineralocorticoid receptor antagonists, digoxin, and nitrates).

## Data Availability

The datasets generated and/or analyzed during the current study are not publicly available due to confidential issues but are available from the corresponding author upon reasonable request.

## Abbreviations

CVDs: Cardiovascular diseases
HF: Heart failure
ADHF: Acute decompensated heart failure
DR: Diuretic resistance
PROVE: Persian registry of cardiovascular disease
LVEF: Left ventricular ejection fraction
BMI: Body mass index
SBP: Systolic blood pressure
DBP: Diastolic blood pressure
BUN: Blood urea nitrogen
Cr: Creatinine
ACEIs: Angiotensin-converting enzyme inhibitors
ARBs: Angiotensin receptor blockers
SD: Standard deviation
HR: Hazard ratio
OR: Odds ratio
SPSS: Statistical Package for the Social Sciences
CI: Confidence interval.
